# Nonlinear Joint Transform Correlator-Based Encryption System in the Fourier Domain for Simultaneous Authentication of Three Images

**DOI:** 10.3390/s26216675

**Published:** 2026-10-22

**Authors:** Daniel Castilla, Adolfo Montero, Juan M. Vilardy, María S. Millán, Elisabet Pérez-Cabré

**Affiliations:** 1 Grupo de Optoelectrónica y Procesamiento de Señales, Department of Electronics, Universidad Popular del Cesar, Valledupar 200001, Colombia; dcastilla@unicesar.edu.co (D.C.); amariomontero@unicesar.edu.co (A.M.); 2 Grupo de Óptica e Informática, Department of Electronics, Universidad Popular del Cesar, Valledupar 200001, Colombia; 3 Applied Optics and Image Processing Group, Universitat Politècnica de Catalunya · BarcelonaTech, 08222 Terrassa, Spain; m.millan@upc.edu (M.S.M.); elisabet.perez@upc.edu (E.P.-C.)

**Keywords:** image authentication, encryption, decryption, joint transform correlator (JTC), phase encoding, Fourier domain, nonlinear signal processing

## Abstract

With the aim of simultaneously authenticating three images, an encryption–decryption system based on a nonlinear Joint Transform Correlator (JTC) in the Fourier domain is proposed. At first, the images to authenticate may be either related to or independent of one another. The input plane of the encryption system is fully phase encoded and includes one primary image to encrypt, four random phase masks (RPMs), and three phase-only distributions containing the information of the three images to be thereafter authenticated. These latter seven phase-only distributions serve as the security keys of the proposed encryption system. The encrypted output is a real-valued distribution derived from two new nonlinear modifications of the joint power spectrum (JPS), which improves resistance to direct inversion and plaintext-based inference. In the decryption system, authentication and image recovery are jointly performed: when all seven security keys are correct, the decrypted image preserves the information that enables the successful simultaneous authentication of the three images due to the two new nonlinear modifications of the JPS; otherwise, the retrieved distribution becomes noise-like. Unlike the nonlinear JTC previously reported for two-image authentication, adding a third authentication distribution generates additional JPS terms that prevent correct recovery of the primary image during decryption, even when all keys match, ultimately precluding simultaneous authentication of the three images. The two new nonlinear JPS modifications overcome this limitation for correct-key decryption. A key-space analysis confirms strong resistance to brute-force attacks, and robustness tests under additive noise, multiplicative noise, and partial occlusion for the encrypted signal are incorporated to assess practical performance. Numerical results indicate that the proposed architecture preserves reliable authentication under moderate degradation of the encrypted distribution. These results support the use of nonlinear JTC architectures as a practical framework for optical multi-image authentication and secure image encryption.

## 1. Introduction

Image authentication is a relevant task in secure information processing because it validates whether a given image carries legitimate and untampered information [1,2,3,4]. In optical security, authentication can be combined with image encryption, particularly through double random phase encoding (DRPE) [5], in order to strengthen confidentiality and access control simultaneously [1,3,6,7,8,9]. Classical DRPE implementations can be developed in an optical 4f-processor [10] or in a joint transform correlator (JTC) architecture [11], where the latter has shown practical advantages thanks to the integration of nonlinear operations [12,13] in different optical processing domains (Fourier [14,15,16], Fresnel [17,18,19,20], fractional Fourier [21,22,23,24], and Gyrator [25,26,27]) to increase cryptographic robustness.

In recent nonlinear JTC-based schemes, the encrypted distribution is derived from a modified joint power spectrum (JPS), so the recovered plaintext becomes strongly sensitive to key mismatch and the system gains resistance against direct inversion strategies [25,28]. This behavior is particularly appropriate for authentication because small perturbations in phase-mask security keys rapidly degrade valid decryption. In parallel, several optical multiple-image authentication approaches have combined image encryption, phase-mask multiplexing, correlation-based verification, and photon-counting strategies for simultaneous recognition tasks [7,8], whereas distortion-invariant encrypted ID-tag methods introduced robustness against scale, rotation, cropping, and noise and can be incorporated as complementary stages in practical verification frameworks [9]. Furthermore, transform-domain extensions and experimental implementations have confirmed that nonlinear optical processing preserves practical feasibility while increasing security margins in realistic cryptographic scenarios [12,16,24,25]. In addition, recent DRPE-oriented studies report improvements in data efficiency and in the treatment of phase singularities, which further motivate robust architectures for secure image recovery and validation [29,30]. A representative system for two-image validation is the nonlinear JTC scheme for the simultaneous authentication of two users, in which two biometric phase distributions act as coupled security keys and successful authentication is achieved only under correct-key decryption [6]. The same study reported a clear discrimination between authorized and unauthorized users through QR code-based verification, thus providing a direct conceptual basis for extending nonlinear JTC authentication toward three-image scenarios [6]. Importantly, this nonlinear JTC formulation for the simultaneous authentication of two users (images) cannot be directly generalized by merely incorporating a third authentication distribution, because the interactions among the three phase-encoded authentication distributions generate additional mutual JPS terms that are neither completely removed during encryption by the nonlinear JPS modification defined in [6] nor canceled during the subsequent decryption process. Consequently, the original primary image cannot be correctly recovered at the output of the decryption system even when all security keys match, which ultimately prevents the simultaneous authentication of the three images. Therefore, simultaneous authentication of three images requires a reformulation of the nonlinear JPS processing and the corresponding decryption algorithm, rather than the direct addition of another authentication image and its associated RPM. Finally, optical triple random-phase encryption has also been reported as an alternative route for reinforcing layered optical protection [31]. Its three-mask architecture is conceptually consistent with the use of additional independent phase keys to enlarge the effective security space in advanced encryption-authentication designs [31].

Recent reviews of optical information-processing architectures identify optical correlators, including the JTC, as relevant platforms for high-speed pattern recognition and optical security, while phase encoding, nonlinear processing, and hybrid optical–digital implementations provide complementary strategies for increasing robustness and practical flexibility [32]. Within multi-user optical security, Zhao and Chi proposed a JTC-based system in which fingerprint-derived phase distributions support identity authentication and flexible group-based or hierarchical access control; however, authentication is performed before decryption, and the scheme does not provide the simultaneous verification of three or more phase-encoded images through several coupled security keys and nonlinear JPS modifications [33]. Liu et al. demonstrated the parallel encryption of multiple image channels into a single encrypted distribution, with cross-talk suppressed by optimized phase masks and spatial separation of the corresponding JPS regions, which also facilitates compressed ciphertext storage and optical implementation without additional hardware; nevertheless, their method is directed toward parallel encryption and selective image recovery rather than simultaneous multi-image authentication, and it does not require the joint correctness of three phase-encoded images during decryption [34]. Xue et al. combined DRPE with compressive sensing to perform the batch authentication, encryption, and compression of multiple images, thereby reducing the amount of authentication information and improving authentication efficiency; however, their method requires separate sampling, encryption-then-compression, carrier-image embedding, reconstruction, and verification stages and produces multiple ciphertext images rather than a single real-valued encrypted image whose correct decryption simultaneously authenticates several images [35]. Zeng et al. proposed an optical multiple-image authentication method based on Fourier single-pixel imaging, sparse spatial-frequency sampling, and multiple logistic maps, achieving authentication at sampling ratios below 5% of the Nyquist limit with numerical and experimental validation; nevertheless, the reconstructed objects do not provide clear visual recovery at such low sampling ratios, and the method requires a set of structured-illumination measurements followed by nonlinear-correlation verification rather than integrating the simultaneous verification of several phase-encoded images into a single nonlinear decryption processor [36]. In addition, Wang et al. proposed a multi-image encryption system based on chaotic fingerprint phase masks and multi-slice diffractive imaging, in which biometric and chaotic parameters facilitate key storage and management, while speckle illumination and different free-space propagation distances provide additional security parameters; however, the method generates a series of noise-like diffractive encrypted patterns and requires an iterative phase-retrieval algorithm for image reconstruction, and it performs multi-image encryption rather than simultaneous authentication through a single real-valued encrypted image and a nonlinear JTC decryption process [37]. From a complementary digital-security perspective, Gao et al. proposed the selective encryption of temporally significant video segments using a discrete sinusoidal memristive Rulkov-neuron map, reducing the encryption time by approximately 90% while preserving strong pseudorandomness and robustness; although this approach supports the broader use of nonlinear transformations for the efficient protection of visual information, it operates in the digital temporal domain and does not perform optical multi-image authentication through coupled phase-encoded security keys [38]. Wang et al. introduced a mask-guided Mamba framework that fuses visible and infrared aerial images by extracting cross-modal guidance and mapping complementary features into a shared hidden state, demonstrating the benefit of jointly processing related images acquired from different sensing modalities; however, its purpose is vehicle detection rather than information security, and it neither encrypts the input images nor verifies their authenticity [39]. Taken together, these works demonstrate significant advances in multi-user access control, parallel and compressed multi-image encryption, reduced-measurement authentication, biometric key management, nonlinear multimedia protection, and the joint processing of multimodal images. However, to the best of our knowledge, none of the reviewed approaches provides a unified encryption–decryption and authentication architecture in which several images are simultaneously verified through a JTC in the FD, several coupled security keys, two new nonlinear modifications of the JPS, and a single real-valued encrypted image.

Motivated by the limitations identified above and by the need to move from two-image to broader multi-image validation to address stricter validation demands in optical cryptography, this work introduces a nonlinear JTC architecture in the Fourier domain (FD) for the simultaneous authentication of three images within a single encryption–decryption workflow. Compared with multifactor authentication approaches and prior nonlinear two-image validation schemes [6,7], the proposed system advances from five to seven keys (four RPMs and three phase-only image distributions), substantially enlarging the effective key space and reinforcing cross-dependence among credentials. The main novelty of this work is the introduction of two new nonlinear modifications of the JPS based on the subtraction of two specific intensity distributions and the three-component normalization term, designed to intensify key-mismatch sensitivity and strengthen resistance to direct inversion and plaintext-oriented attacks while preserving a single real-valued ciphertext suitable for transmission. In addition, a third authentication image is incorporated without sequential encryption stages, and the two new nonlinear JPS modifications enable the simultaneous authentication of the three images only when all phase-only security keys are jointly correct and the embedded binary authentication payload is correctly recovered. In contrast to the two-image simultaneous authentication system of Ref. [6], the proposed architecture is not obtained by simply incorporating an additional authentication distribution, since the two nonlinear modifications of the JPS proposed in this work overcome the limitation that arises when the formulation of Ref. [6] is extended to three images to be authenticated, thereby ensuring the correct recovery of the primary image during decryption when all security keys match and, consequently, the simultaneous authentication of the three images. The manuscript then presents the full description of the encryption and decryption-authentication systems in Section 2 and evaluates their performance through numerical results, security scaling, and robustness under additive noise, multiplicative perturbations, and partial occlusion of the encrypted distribution in Section 3. Finally, the conclusions are summarized in Section 4.

## 2. Encryption, Decryption, and Authentication with a Nonlinear JTC in the FD for the Simultaneous Authentication of Three Images

### 2.1. Encryption System

**Figure 1:**
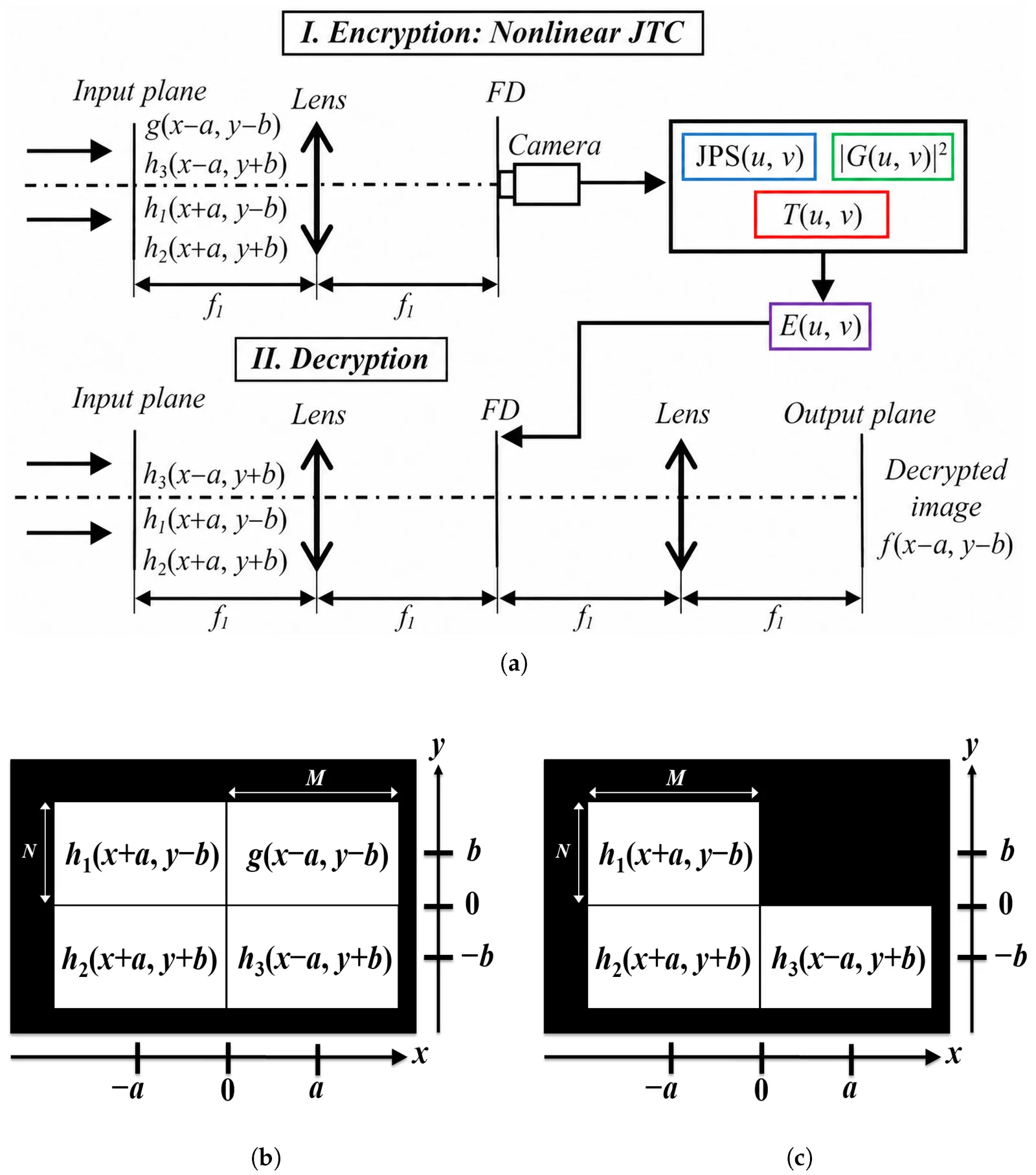
(**a**) Complete optical setup of the proposed security system, where part I corresponds to the encryption system implemented by a nonlinear JTC architecture and part II corresponds to the decryption system carried out by a 4*f*-processor based on two successive Fourier transforms. Input-plane distributions used in the (**b**) encryption system and in the (**c**) decryption system.

The primary image to encrypt f(x,y) is a binary image that contains all the information required to carry out the simultaneous authentication process of the three images. Four statistically independent RPMs are defined in the spatial domain, denoted by the coordinates *x* and *y*, as follows [5]:

(1) $$\begin{array}{c} r\left(x,y\right)=\exp\left\{i\pi s\left(x,y\right)\right\},\;k_{m}\left(x,y\right)=\exp\left\{i\pi n_{m}\left(x,y\right)\right\},\;m\in \left\{1,2,3\right\}, \end{array}$$

where s(x,y) and $n_{m}\left(x,y\right)$ are uniformly distributed random functions with values in the interval of [0,1]. Each function involved in the encryption and decryption systems is represented by an image of M×N pixels. Figure 1a depicts the complete optical setup for the proposed security system, where part I corresponds to the encryption system implemented with a nonlinear JTC architecture, and part II corresponds to the decryption system carried out by a 4*f*-processor.

Figure 1b shows the locations of the four data distributions in the input plane of the JTC. The primary image to encrypt f(x,y) is phase encoded $f_{ph}\left(x,y\right)=\exp\left\{i\pi f\left(x,y\right)\right\}$ and bonded to the RPM r(x,y), in order to obtain the first data distribution g(x,y) of the input plane of the encryption system [40]:

(2) $$\begin{array}{c} g\left(x,y\right)=f_{ph}\left(x,y\right)r\left(x,y\right)=\exp\left\{i\pi \left[f\left(x,y\right)+s\left(x,y\right)\right]\right\}. \end{array}$$

The other three data distributions in the input plane of the JTC correspond to the functions $h_{m}\left(x,y\right)$, which are given by the product of an RPM $k_{m}\left(x,y\right)$ and one of the images to authenticate $p_{m}\left(x,y\right)$ encoded in phase

(3) $$\begin{array}{c} h_{m}\left(x,y\right)=\exp\left\{i\pi p_{m}\left(x,y\right)\right\}k_{m}\left(x,y\right)=\exp\left\{i\pi \left[p_{m}\left(x,y\right)+n_{m}\left(x,y\right)\right]\right\}. \end{array}$$

The three images that will later be authenticated may be related to one another, as they may belong to or be captured from the same person or animal or from a material object, or they may be independent of one another. The first data distribution g(x,y) is spatially shifted in the JTC input plane centred at the coordinates (x,y)=(a,b). The second, third and fourth data distributions of the input plane of the JTC denoted by the functions $h_{1}\left(x,y\right)$, $h_{2}\left(x,y\right)$, and $h_{3}\left(x,y\right)$ are centred at coordinates (x,y)=(−a,b), (x,y)=(−a,−b), and (x,y)=(a,−b), respectively. All these four phase-only data distributions are tiled adjacently without spatial overlap or interstitial gaps. The joint power spectrum (JPS) is the intensity of the Fourier Transform (FT) of the input plane of the JTC and is given by

(4) $$\begin{array}{cc} \mathrm{JPS}(u,v)= & {\left|F\left\{g\left(x-a,y-b\right)+h_{1}\left(x+a,y-b\right)+h_{2}\left(x+a,y+b\right)+h_{3}\left(x-a,y+b\right)\right\}\right|}^{2}\\ = & |G\left(u,v\right)e^{-i2\pi (au+bv)}+H_{1}\left(u,v\right)e^{-i2\pi (-au+bv)}+H_{2}\left(u,v\right)e^{-i2\pi (-au-bv)}\\ & +H_{3}\left(u,v\right)e^{-i2\pi (au-bv)}{|}^{2}, \end{array}$$

where *u* and *v* denote the spatial-frequency coordinates, and each function written in uppercase corresponds to the FT of its lowercase counterpart.

Finally, the encrypted image E(u,v) is obtained when two new nonlinear modifications are introduced over the JPS

(5) $$\begin{array}{cc} E(u,v)= & \frac{\mathrm{JPS}\left(u,v\right)-\left[{\left|G\left(u,v\right)\right|}^{2}+T\left(u,v\right)\right]}{T(u,v)}\\ = & \frac{1}{T(u,v)}[G\left(u,v\right)H_{1}^{*}\left(u,v\right)e^{-i2\pi (2au)}+G\left(u,v\right)H_{2}^{*}\left(u,v\right)e^{-i2\pi (2au+2bv)}\\ & +G\left(u,v\right)H_{3}^{*}\left(u,v\right)e^{-i2\pi (2bv)}+G^{*}\left(u,v\right)H_{1}\left(u,v\right)e^{-i2\pi (-2au)}\\ & +G^{*}\left(u,v\right)H_{2}\left(u,v\right)e^{-i2\pi (-2au-2bv)}+G^{*}\left(u,v\right)H_{3}\left(u,v\right)e^{-i2\pi (-2bv)}], \end{array}$$

where

(6) $$\begin{array}{c} T\left(u,v\right)={\left|F\{h_{1}\left(x+a,y-b\right)+h_{2}\left(x+a,y+b\right)+h_{3}\left(x-a,y+b\right)\}\right|}^{2}, \end{array}$$

and the complex conjugation operation is denoted by the superscript * in Equation (5). If T(u,v) is equal to zero at a particular pair of spatial-frequency coordinates (u,v), the corresponding intensity value is replaced by a small positive constant to avoid singularities when computing E(u,v), following the numerical procedure reported in Ref. [13]. In a practical hybrid optoelectronic realization, very small nonzero values of T(u,v) would additionally require a detector-dependent denominator floor and output clipping to limit noise amplification caused by finite dynamic range and quantization. These hardware-dependent stabilization parameters were not modeled in the present numerical proof of concept. The two new nonlinear modifications of the JPS, given by the subtraction of the terms $\left[{\left|G\left(u,v\right)\right|}^{2}+T\left(u,v\right)\right]$ and the division by T(u,v), are totally different from the modifications of the JPS presented in [6]. These two new nonlinear modifications of the JPS are incorporated into the definition of the encrypted image with a twofold objective: to enable the correct recovery of the primary image when the three authentication distributions and the remaining security keys are correct, and to strengthen the security of the encrypted image against plaintext attacks, consistent with the nonlinear security approaches analyzed in the Fourier [13], Fresnel [17], and Gyrator [25] domains. The cross-correlation terms in the FD between G(u,v) and $H_{m}\left(u,v\right)$ are obtained in Equation (5) due to the subtraction of the terms $\left[{\left|G\left(u,v\right)\right|}^{2}+T\left(u,v\right)\right]$ from the JPS. Note that T(u,v) and $\left[{\left|G\left(u,v\right)\right|}^{2}+T\left(u,v\right)\right]$ are intensity distributions (real-valued terms), which is a very important property for an experimental implementation of the nonlinear DRPE [5,12]. The subtraction of the terms $\left[{\left|G\left(u,v\right)\right|}^{2}+T\left(u,v\right)\right]$ removes from the JPS the self-intensity term of G(u,v) and the intensity contributions contained in T(u,v), including the self-intensity and mutual cross-correlation terms associated with the three authentication distributions, whereas division by T(u,v) introduces a signal- and key-dependent normalization. Consequently, the encrypted image cannot be expressed through the standard linear Fourier-domain input–output relationship exploited by conventional direct-inversion and plaintext-attack procedures. This nonlinear processing has been previously demonstrated and discussed for JTC encryption systems through intensity-dependent normalization in the Fourier domain [13], combined nonzero-order processing and normalization in the fractional Fourier domain [21], and nonlinear JPS modifications for double-image encryption in the Fourier domain [28].

In this work, the modifications of the JPS given in Equation (5) are performed by digital computation. The encrypted image E(u,v) is a real-valued function computed from the three intensity distributions JPS(u,v), ${\left|G\left(u,v\right)\right|}^{2}$, and T(u,v). The proposed security system increases the number of authentication images by one relative to the previous proposal [6], while the number of intensity distributions required to generate the encrypted function is reduced by one. In a prospective hybrid optoelectronic realization, these three intensity distributions could, in principle, be acquired sequentially with a camera by displaying the corresponding data distributions at the input plane of a JTC. The seven security keys of the encryption system are represented by the four RPMs, r(x,y) and $k_{m}\left(x,y\right)$ with m=1,2,3, and the three images to authenticate, $p_{m}\left(x,y\right)$ with m=1,2,3. The optical Fourier-transform stages depicted in Figure 1a could be implemented using a 2*f* JTC processor for encryption and a 4*f* processor for decryption. During each optical propagation, the spatial information carried by all pixels of the input wavefront is processed simultaneously and in parallel, and the corresponding Fourier-domain intensity distribution can be optically formed as the wavefront propagates at the speed of light [1,2,3,4,10,12]. This speed-of-light property refers specifically to optical propagation and parallel Fourier transformation; the sequential display of the input distributions, camera exposure and readout, data transfer, and digital computation of the nonlinear encrypted distribution remain limited by the response times of the corresponding electronic devices. Therefore, the nonlinear construction of E(u,v) remains a digital operation. This discussion describes a possible hybrid optoelectronic implementation pathway and does not represent an experimental system constructed or validated in the present work.

A practical implementation would additionally require evaluating spatial-light-modulator sampling and quantization, finite aperture, optical misalignment, phase-modulation errors, detector noise, camera quantization, sequential-acquisition inconsistencies, and finite dynamic range. These effects could alter the measured intensity distributions used to compute E(u,v), causing an encrypted distribution generated from experimental measurements to deviate from the ideal encrypted image defined by Equation (5). Together with the detector-dependent denominator stabilization described after Equation (6), their influence on encrypted-image generation would require experimental characterization, calibration, and compensation. These hardware-dependent effects were not modeled in the present numerical study, and their impact on subsequent decryption and authentication performance remains to be evaluated in a future experimental implementation.

### 2.2. Decryption System and Authentication Process

The decryption system utilizes a 4*f*-processor to perform two sequential Fourier transforms (Figure 1a, part II). The three images to be authenticated must be provided in situ to the input plane of the decryption system in order to be compared with the information contained in the encrypted image. Let us assume that the input plane of the decryption system is composed by the three phase-only distributions $h_{1}\left(x,y\right)$, $h_{2}\left(x,y\right)$ and $h_{3}\left(x,y\right)$ centred at coordinates (x,y)=(−a,b), (x,y)=(−a,−b) and (x,y)=(a,−b), respectively (Figure 1c), which coincide with the positions already used for encryption (Figure 1b); these three phase-only distributions contain the three RPMs $k_{m}\left(x,y\right)$ (m=1,2,3) and the three images to authenticate ($p_{m}\left(x,y\right)$ with m=1,2,3). The first step of the decryption system is the product between the FT of the input plane of the decryption system and the encrypted image E(u,v) (Equation (5)):

(7) $$\begin{array}{cc} D(u,v)= & F\{h_{1}\left(x+a,y-b\right)+h_{2}\left(x+a,y+b\right)+h_{3}\left(x-a,y+b\right)\}E\left(u,v\right)\\ = & \left[H_{1}\left(u,v\right)e^{-i2\pi (-au+bv)}+H_{2}\left(u,v\right)e^{-i2\pi (-au-bv)}+H_{3}\left(u,v\right)e^{-i2\pi (au-bv)}\right]E\left(u,v\right)\\ = & \frac{1}{T(u,v)}[G\left(u,v\right){\left|H_{1}\left(u,v\right)\right|}^{2}e^{-i2\pi (au+bv)}+G\left(u,v\right)H_{1}\left(u,v\right)H_{2}^{*}\left(u,v\right)e^{-i2\pi (au+3bv)}\\ & +G\left(u,v\right)H_{1}\left(u,v\right)H_{3}^{*}\left(u,v\right)e^{-i2\pi (-au+3bv)}+G^{*}\left(u,v\right)H_{1}^{2}\left(u,v\right)e^{-i2\pi (-3au+bv)}\\ & +G^{*}\left(u,v\right)H_{1}\left(u,v\right)H_{2}\left(u,v\right)e^{-i2\pi (-3au-bv)}+G^{*}\left(u,v\right)H_{1}\left(u,v\right)H_{3}\left(u,v\right)e^{-i2\pi (-au-bv)}\\ & +G\left(u,v\right)H_{1}^{*}\left(u,v\right)H_{2}\left(u,v\right)e^{-i2\pi (au-bv)}+G\left(u,v\right){\left|H_{2}\left(u,v\right)\right|}^{2}e^{-i2\pi (au+bv)}\\ & +G\left(u,v\right)H_{2}\left(u,v\right)H_{3}^{*}\left(u,v\right)e^{-i2\pi (-au+bv)}+G^{*}\left(u,v\right)H_{1}\left(u,v\right)H_{2}\left(u,v\right)e^{-i2\pi (-3au-bv)}\\ & +G^{*}\left(u,v\right)H_{2}^{2}\left(u,v\right)e^{-i2\pi (-3au-3bv)}+G^{*}\left(u,v\right)H_{2}\left(u,v\right)H_{3}\left(u,v\right)e^{-i2\pi (-au-3bv)}\\ & +G\left(u,v\right)H_{1}^{*}\left(u,v\right)H_{3}\left(u,v\right)e^{-i2\pi (3au-bv)}+G\left(u,v\right)H_{2}^{*}\left(u,v\right)H_{3}\left(u,v\right)e^{-i2\pi (3au+bv)}\\ & +G\left(u,v\right){\left|H_{3}\left(u,v\right)\right|}^{2}e^{-i2\pi (au+bv)}+G^{*}\left(u,v\right)H_{1}\left(u,v\right)H_{3}\left(u,v\right)e^{-i2\pi (-au-bv)}\\ & +G^{*}\left(u,v\right)H_{2}\left(u,v\right)H_{3}\left(u,v\right)e^{-i2\pi (-au-3bv)}+G^{*}\left(u,v\right)H_{3}^{2}\left(u,v\right)e^{-i2\pi (au-3bv)}]. \end{array}$$

The output of the decryption system is given by the inverse FT of Equation (7) and consists of several spatially separated distributions. The first, second, third, seventh, eighth, ninth, thirteenth, fourteenth, and fifteenth terms of Equation (7) contain all the information necessary to recover the data distribution $\hat{g}\left(x,y\right)$ centred at coordinates (x,y)=(a,b). The other nine remaining terms of Equation (7) are spatially separated from the data distribution $\hat{g}\left(x,y\right)$ at the output plane of the decryption system and contain noise-like data distributions. The data distribution $\hat{g}\left(x,y\right)$ recovered from Equation (7) is given by

(8) $$\begin{array}{cc} \hat{g}\left(x-a,y-b\right)= & F^{-1}\{\frac{G\left(u,v\right)e^{-i2\pi (au+bv)}}{T(u,v)}[{\left|H_{1}\left(u,v\right)\right|}^{2}+{\left|H_{2}\left(u,v\right)\right|}^{2}+{\left|H_{3}\left(u,v\right)\right|}^{2}\\ & +H_{1}\left(u,v\right)H_{2}^{*}\left(u,v\right)e^{-i2\pi (2bv)}+H_{1}\left(u,v\right)H_{3}^{*}\left(u,v\right)e^{-i2\pi (-2au+2bv)}\\ & +H_{1}^{*}\left(u,v\right)H_{2}\left(u,v\right)e^{-i2\pi (-2bv)}+H_{2}\left(u,v\right)H_{3}^{*}\left(u,v\right)e^{-i2\pi (-2au)}\\ & +H_{1}^{*}\left(u,v\right)H_{3}\left(u,v\right)e^{-i2\pi (2au-2bv)}+H_{2}^{*}\left(u,v\right)H_{3}\left(u,v\right)e^{-i2\pi (2au)}]\}. \end{array}$$

The mathematical difference between the proposed system and the two-image formulation of Ref. [6] becomes evident in Equation (8). When all security keys used during decryption match those employed during encryption, the complete expression enclosed in square brackets in Equation (8) is exactly equal to the three-component intensity distribution T(u,v) defined in Equation (6). Therefore, this factor is canceled by the denominator T(u,v), leaving the spatially shifted spectrum of the primary encoded image G(u,v), from which the primary image can be correctly recovered. This exact cancellation depends on the subtraction and three-component normalization introduced in Equation (5). If the nonlinear modifications defined in Ref. [6] were directly extended by merely adding a third authentication distribution, the additional intensity and mutual JPS terms involving $H_{3}\left(u,v\right)$ would not be completely canceled during decryption, and the primary image could not be correctly recovered even when all security keys matched. Thus, the proposed nonlinear JPS modifications constitute a necessary mathematical reformulation for the simultaneous authentication of three images rather than a direct numerical extension of the previous two-image system.

The recovered primary image $\hat{f}\left(x,y\right)$ estimate is obtained from phase extraction applied to the product between the result of Equation (8) and the complex conjugate of the RPM r(x,y) centred at coordinates (x,y)=(a,b):

(9) $$\begin{array}{c} \pi \hat{f}\left(x-a,y-b\right)=\arg\left\{\hat{g}\left(x-a,y-b\right)r^{*}\left(x-a,y-b\right)\right\}, \end{array}$$

where $\hat{f}\left(x,y\right)$ is the decrypted image and arg denotes the phase of a complex-valued function. Proper recovery of the primary image f(x,y) as the decrypted image $\hat{f}\left(x,y\right)$ is achieved when the seven security keys (the four RPMs r(x,y) and $k_{m}\left(x,y\right)$, and the three images to authenticate $p_{m}\left(x,y\right)$) used in the decryption system are the same security keys utilized in the encryption system. The two new nonlinear modifications of the JPS introduced in Equation (5) are essential in order to allow the correct recovery of the primary image that was encrypted.

The authentication process is performed by evaluating the decrypted image against the primary image of the encryption system. The simultaneous authentication of the three images encoded in phase and placed at the input plane of the decryption system is successful when the decrypted image is a replica of the primary image of the encryption system. Therefore, decryption and authentication are intrinsically linked. If one or more security keys are incorrect in the decryption system, the recovered distribution for the decrypted image would be noisy, and authentication would fail.

The loss of correct primary-image recovery and the consequent authentication failure described above arise from the distinct but complementary mathematical roles of the seven security keys. Six of these keys form three coupled phase-only distributions: as defined in Equation (3), each distribution $h_{m}\left(x,y\right)$ combines one image to authenticate $p_{m}\left(x,y\right)$ with its corresponding RPM $k_{m}\left(x,y\right)$. When the three key pairs supplied to the decryption system match those employed during encryption, the complete Fourier-domain factor enclosed in square brackets in Equation (8) reproduces the three-component intensity distribution T(u,v) defined in Equation (6). Consequently, this factor is exactly canceled by the denominator T(u,v), allowing the primary encoded distribution to be correctly recovered. If either $p_{m}\left(x,y\right)$ or $k_{m}\left(x,y\right)$ does not match its encryption counterpart, the corresponding $H_{m}\left(u,v\right)$ used during decryption is altered and this exact cancellation condition is no longer satisfied. The seventh security key, r(x,y), performs a different but complementary function in the final phase-decoding operation of Equation (9). An incorrect r(x,y) does not properly compensate the phase mask bonded to the primary image during encryption, even when the other six security keys are correct. Therefore, the six keys grouped into the three coupled authentication distributions and the seventh phase-decoding key are jointly required to guarantee exact recovery of the primary image and successful simultaneous authentication of the three images.

## 3. Numerical Simulations and Results

**Figure 2:**
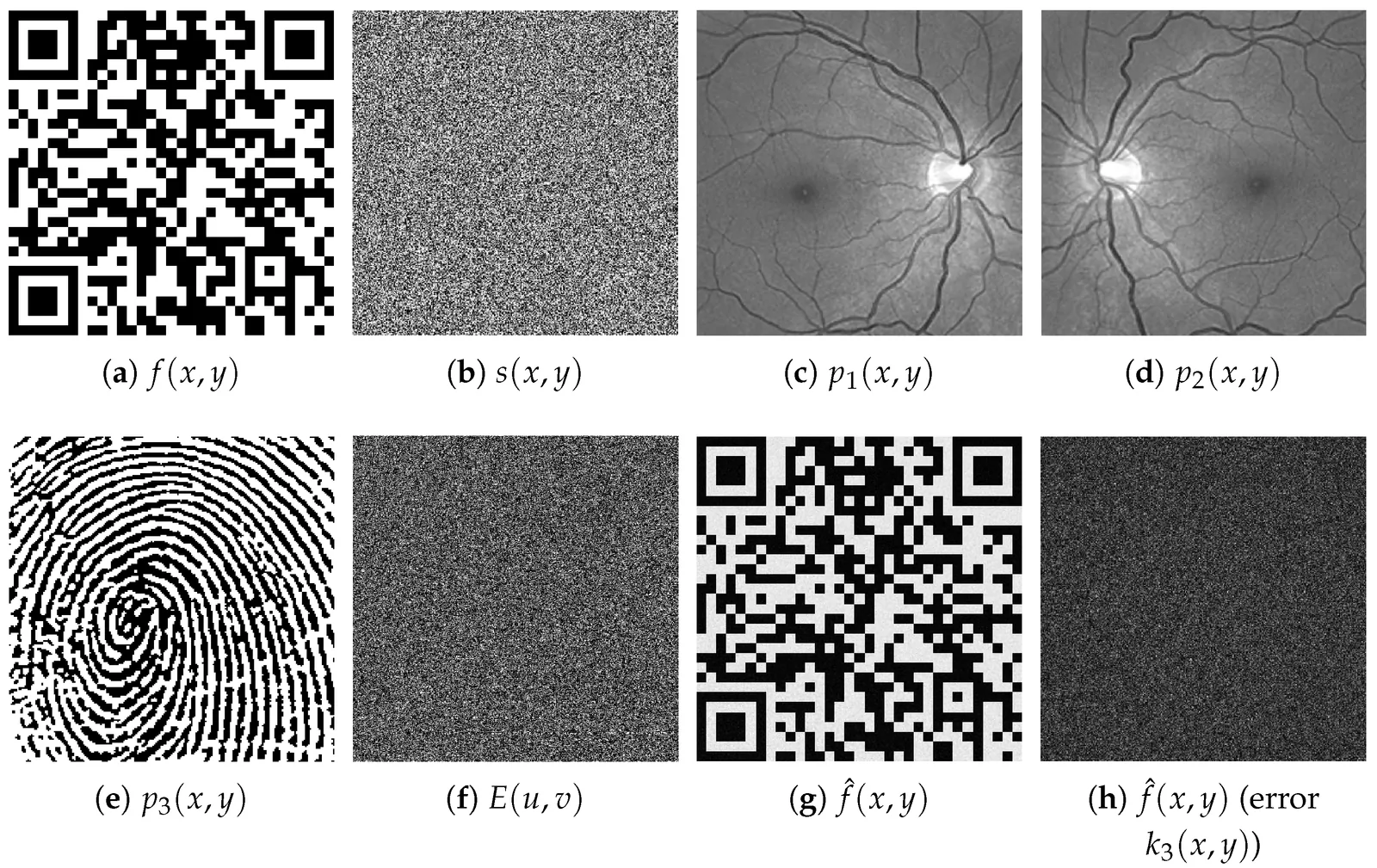
(**a**) Primary image to encrypt f(x,y). (**b**) Random code s(x,y) of the RPM r(x,y). Three biometric images to authenticate: (**c**) $p_{1}\left(x,y\right)$, (**d**) $p_{2}\left(x,y\right)$, and (**e**) $p_{3}\left(x,y\right)$. (**f**) Real-valued encrypted image E(u,v). (**g**) Correctly decrypted image $\hat{f}\left(x,y\right)$ obtained with the seven valid security keys. (**h**) Incorrectly decrypted image obtained when the security key given by the RPM $k_{3}\left(x,y\right)$ is wrong.

Numerical simulations are presented for the encryption–decryption system and authentication process described in Section 2. All the grayscale images have a resolution of 300×300 pixels. Figure 2 displays the results corresponding to the proposed security system. Figure 2a,b shows the primary image to encrypt f(x,y) and the random code s(x,y) associated with the RPM r(x,y), respectively. The grayscale patterns associated with the random functions $n_{m}\left(x,y\right)$ of the RPMs $k_{m}\left(x,y\right)$ are different from one another, although their visual appearance is similar to that shown in Figure 2b. The three images to subsequently authenticate, $p_{1}\left(x,y\right)$, $p_{2}\left(x,y\right)$, and $p_{3}\left(x,y\right)$, are shown in Figure 2c, d, and e, respectively. The two gray-level eye-fundus images shown in Figure 2c,d and the fingerprint image shown in Figure 2e were acquired directly by the authors from a single member of the author group using commercially available imaging devices. These images were not obtained from a public database, external dataset, or previously published source. They were processed into the 300×300-pixel grayscale format and used exclusively as representative exact digital-image distributions in the numerical simulations. No individuals outside the author group were recruited, and no clinical evaluation, diagnosis, biometric feature extraction, or practical biometric-recognition analysis was performed. The real-valued encrypted image E(u,v) is a noisy pattern presented in Figure 2f.

When the seven security keys (the four RPMs r(x,y) and $k_{m}\left(x,y\right)$, and the three images to authenticate $p_{m}\left(x,y\right)$) used in the decryption system are identical to those employed in the encryption system, the correctly recovered image $\hat{f}\left(x,y\right)$ shown in Figure 2g is obtained. This image corresponds to the decrypted image and is a replica of the primary image presented in Figure 2a. Although the recovered primary image shown in Figure 2g closely reproduces the original binary QR image, its pixel values can exhibit small numerical deviations from the exact binary values of 0 and 1. Small nonzero values of T(u,v) can amplify finite-precision numerical errors during the computation of E(u,v) and contribute to these deviations in the recovered image. Nevertheless, the recovered distribution maintains high fidelity to the original primary image, and the QR-code representation adequately tolerates these small pixel-value variations. Consequently, these numerical deviations do not prevent the satisfactory recovery of the primary image at the output of the decryption system. The incorrectly decrypted image shown in Figure 2h is a noisy signal and corresponds to the use of an incorrect security key in the decryption system, such as the RPM $k_{3}\left(x,y\right)$. The decrypted image $\hat{f}\left(x,y\right)$ will not be equal to the primary image f(x,y) that was encrypted if one or more of the seven security keys used in the decryption system are different from those employed in the encryption system.

The recovery quality of the decrypted image $\hat{f}\left(x,y\right)$ with respect to the original primary image f(x,y) is evaluated using the normalized root mean square error (NRMSE) defined in Equation (10):

(10) $$\begin{array}{c} \mathrm{NRMSE}={\left(\frac{\sum_{x=1}^{M}\sum_{y=1}^{N}{\left[f\left(x,y\right)-\hat{f}\left(x,y\right)\right]}^{2}}{\sum_{x=1}^{M}\sum_{y=1}^{N}{\left[f\left(x,y\right)\right]}^{2}}\right)}^{1/2}, \end{array}$$

with M=N=300, the NRMSE is nonnegative and is equal to zero when the decrypted image exactly reproduces the original primary image. Although this metric is not generally restricted to the interval from 0 to 1, all NRMSE values obtained in the numerical cases evaluated in this work were between 0 and 1. Within these evaluated cases, values close to zero indicate high reconstruction fidelity, whereas larger values indicate increasing degradation of the decrypted image. The NRMSE between the decrypted image shown in Figure 2g and the primary image shown in Figure 2a is 0.087, confirming the high reconstruction fidelity obtained when the seven correct security keys are used in the decryption system. This high reconstruction quality is mainly due to the two new nonlinear modifications applied to the JPS in Equation (5). By contrast, the NRMSE of the incorrectly decrypted image shown in Figure 2h, calculated with respect to the primary image shown in Figure 2a, is 0.81. This value is substantially larger than that obtained with the seven correct security keys and therefore indicates poor recovery quality.

Unlike encryption schemes controlled by scalar-valued parameters, each security key in the proposed system is a complete 300×300-pixel image distribution. Therefore, a key mismatch cannot be represented by a unique scalar variation unless a specific perturbation model and a corresponding distance measure are first defined. Possible models could include phase deviation, progressive pixel replacement, or a prescribed correlation level between the correct and incorrect distributions; each model would produce a different NRMSE response. Furthermore, the seven keys define seven single-key mismatch classes and 120 possible subsets involving two or more mismatched keys, with each subset containing an extremely large number of possible image realizations. Consequently, the NRMSE cannot be uniquely characterized as a function only of the identity or number of incorrect keys, nor is it necessarily expected to vary monotonically with the number of mismatched keys. Figure 2h therefore represents the full replacement of one security key by an independently generated incorrect RPM, rather than a point on a universal one-dimensional key-sensitivity curve. A complete multidimensional sensitivity analysis would require a separately defined perturbation and sampling framework for the RPMs and authentication images and is beyond the scope of the present work.

The simultaneous authentication of the three images, $p_{1}\left(x,y\right)$, $p_{2}\left(x,y\right)$, and $p_{3}\left(x,y\right)$, is successfully achieved when the Quick Response (QR) code embedded in the decrypted image $\hat{f}\left(x,y\right)$ of Figure 2g is read and the retrieved message is *The authentication process has been successful*. By contrast, when the incorrectly decrypted image shown in Figure 2h is read as a QR code, the message *The authentication process has been successful* is not recovered, which indicates that the simultaneous authentication process for the three images has failed. Thus, the NRMSE quantifies the pixel-level reconstruction error, whereas successful or unsuccessful extraction of the expected QR message from the decrypted image provides the complementary task-level criterion for determining the authentication outcome. The retinal and fingerprint images in Figure 2c–e are used as exact digital-image distributions to numerically validate the proposed architecture. Although these images contain biometric information, the proposed system does not perform biometric feature extraction, image registration, alignment, classification, or tolerance-based matching and therefore does not claim practical biometric recognition under intra-class acquisition variability. In this work, successful multi-image authentication means that the three complete digital authentication distributions supplied to the decryption system match those used during encryption, thereby enabling the correct recovery and decoding of the primary QR image.

The proposed security system uses seven security keys: four statistically independent RPMs (r(x,y) and $k_{m}\left(x,y\right)$) that serve as random cryptographic keys, together with three structured security keys represented by the images to authenticate $p_{m}\left(x,y\right)$, which serve as authentication-image credentials. Although the three authentication images are not random cryptographic keys, they function as indispensable security keys within the proposed architecture because each must match its encryption-stage counterpart to enable the correct recovery of the primary image and the simultaneous authentication of the three images. Under the adopted digital representation, each security key has a resolution of 300×300 pixels and each pixel can take 256 possible values. If all seven security keys are considered, the resulting nominal digital key-search space contains $256^{7(300)(300)}=256^{630,000}$ possible combinations. However, because the three authentication-image credentials contain structured information, this value represents a nominal upper-bound enumeration and should not be interpreted as the independent random-key entropy of the system. Importantly, the resistance to exhaustive brute-force attacks does not depend on assigning random entropy to these three structured security keys. Considering only the four statistically independent RPMs, the random cryptographic key space contains $256^{4(300)(300)}=256^{360,000}$ possible combinations. This extremely large random-key space makes an exhaustive search over the four RPMs computationally impractical and provides strong resistance to brute-force attacks under the adopted sampling and quantization model [41]. By comparison, the two-image authentication system reported in Ref. [6] employs three RPMs, corresponding to a random cryptographic key space of $256^{3(300)(300)}=256^{270,000}$ possible combinations under the same digital representation. Therefore, the random-key space of the proposed system is greater by a factor of $256^{90,000}$.

Moreover, the resistance of the proposed encryption–decryption and authentication systems against plaintext attacks is reinforced by the nonlinear operations introduced in this work. In particular, these nonlinear operations correspond to the two new nonlinear modifications applied to the JPS in Equation (5), together with the phase encoding of the primary image and the three images to authenticate. Related precedents include phase-encoded nonlinear JTC encryption of a single image [12], combined nonzero-order processing and normalization in the fractional Fourier domain [21], and nonlinear double-image encryption in the Fourier domain [28]. The threat model adopted in this work assumes that the attacker knows the encryption–decryption architecture and its algorithms and may observe the real-valued encrypted distribution but does not possess the four RPMs or the three exact authentication-image credentials. In this context, direct inversion is closely related to known-plaintext attack (KPA) and chosen-plaintext attack (CPA): these attack models provide plaintext–ciphertext pairs, whereas direct inversion represents the algebraic procedure used to estimate the encryption transformation or isolate its security keys from those pairs [3]. Since previously published KPA and CPA procedures are derived for specific encryption mappings, as illustrated by the Fourier-domain CPA analysis [13], the Fresnel-domain KPA and CPA analyses [17], and the Gyrator-domain plaintext-attack analyses [25], they cannot be transferred unchanged to the proposed architecture. The subtraction of selected intensity terms and the signal- and key-dependent normalization applied to the JPS prevent the straightforward use of the linear isolation procedures employed against conventional linear optical encryption systems. Any new KPA or CPA specifically adapted to the proposed system would have to account for its seven coupled security keys, three phase-encoded authentication distributions, and three-component normalization and would require the formulation of a new architecture-specific cryptanalytic procedure. The present numerical work does not constitute a comprehensive evaluation against every possible ciphertext-only, inference-based, known-plaintext, or chosen-plaintext attack specifically designed for the proposed architecture. The formulation and quantitative validation of such new architecture-specific attacks generally constitute independent cryptanalytic studies and are commonly reported in dedicated subsequent publications because each attack requires an explicit definition of the attacker’s capabilities, available information, access to the encryption system, estimation procedure, and success criterion. Developing and validating these new attacks is therefore beyond the scope of the present paper, which focuses on the formulation and numerical validation of the proposed encryption–decryption and simultaneous authentication system. Accordingly, the security claims presented here are limited to the established direct-inversion, KPA, and CPA procedures previously analyzed for nonlinear JTC encryption systems and do not imply unconditional resistance to every attack that could subsequently be developed specifically for the proposed architecture.

### Occlusion and Noise Tests on the Encrypted Image

In practical optical and digital channels, the encrypted image may be affected by additive noise, multiplicative noise, or partial data loss. This is relevant for real optical implementations where camera sensor noise, finite quantization, and partial data clipping are unavoidable. Let us consider that the encrypted image generated by the encryption system is deliberately degraded by simulated occlusion or noise sources. In such cases, let us evaluate the performance of the decryption system and the authentication outcome [21]. In each case, the resulting decrypted image is compared with the primary image that was originally encrypted by computing the NRMSE between these images, and the success or failure of the simultaneous authentication of the three images is verified by reading the QR code recovered from the new decrypted image.

**Figure 3:**
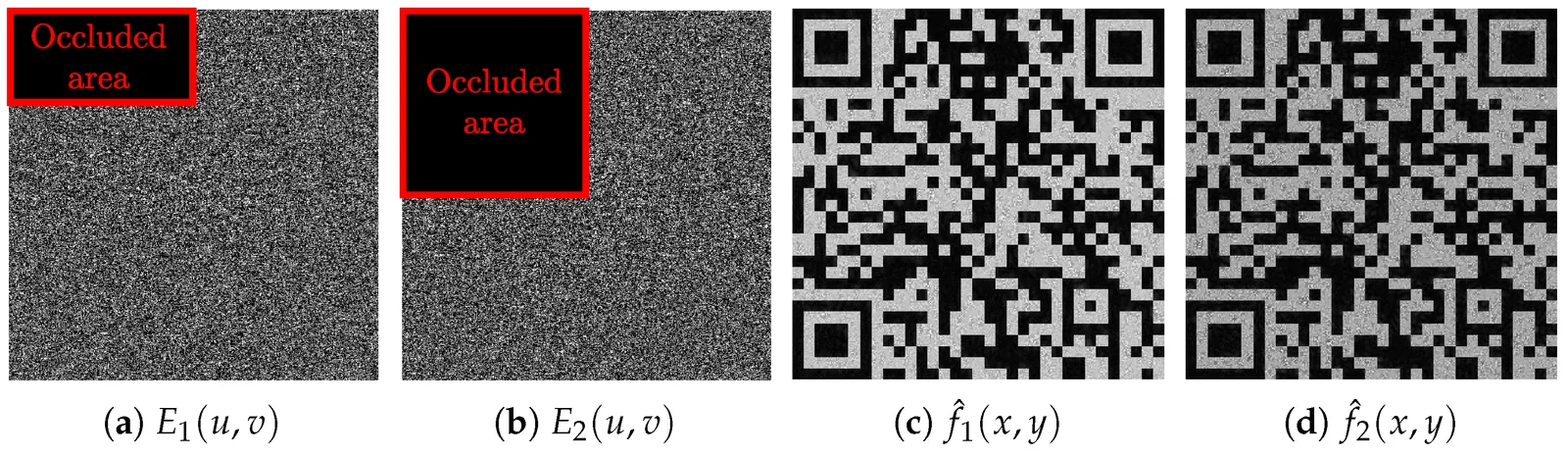
Encrypted images with partial occlusion over the following fractions of their total area: (**a**) 12.5% and (**b**) 25%. Corresponding decrypted images obtained from the occluded encrypted images of Figure 3a and Figure 3b: (**c**) and (**d**), respectively.

The encrypted image of Figure 2f is partially occluded over 12.5% (Figure 3a) and 25% (Figure 3b) of its total area, where the occluded pixels are replaced by zero-valued pixels (black pixels) and their corresponding decrypted images shown in Figure 3c and Figure 3d are obtained, respectively, when the seven correct security keys are used. The NRMSE values between the primary image of Figure 2a and the decrypted images of Figure 3c and Figure 3d are 0.2654 and 0.3743, respectively. As expected, the larger the occluded area of the encrypted image, the more degraded the corresponding decrypted image becomes.

Additional simulations with occlusion ratios greater than 25% were performed to determine the authentication-failure threshold associated with partial occlusion of the encrypted image. This threshold, defined as the lowest tested occlusion ratio at which no text could be extracted from the recovered QR code using a smartphone camera, was found at 37%. At this occlusion ratio, the NRMSE between the original primary image f(x,y) and the decrypted image $\hat{f}\left(x,y\right)$ increased to 0.5728. At this threshold, the decrypted image did not preserve sufficient QR-code information for text extraction; therefore, the simultaneous authentication of the three images failed.

**Figure 4:**
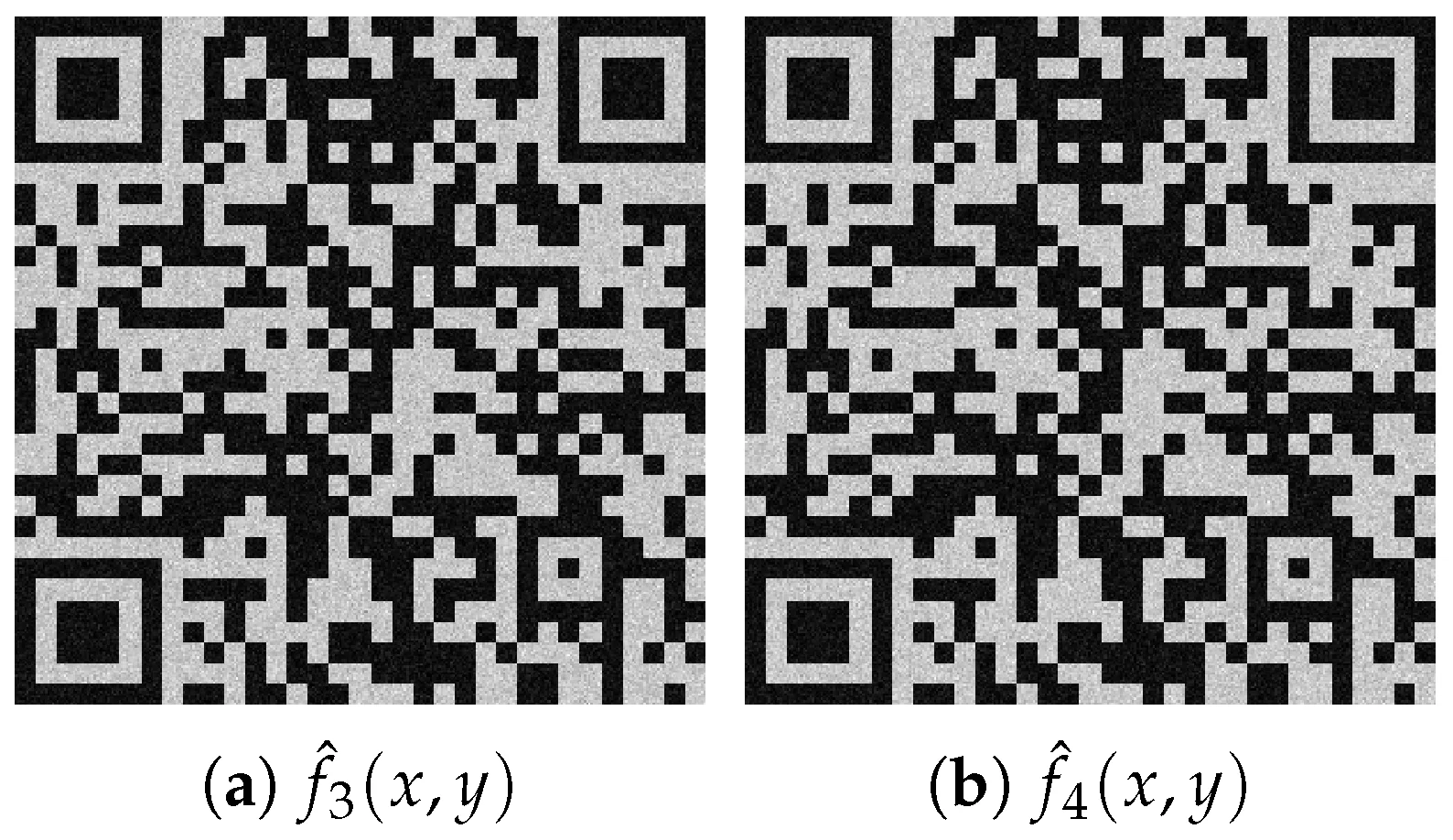
Decrypted images obtained when the encrypted image of Figure 2f is degraded by zero-mean Gaussian white noise with variance $\sigma^{2}=0.2$: (**a**) additive noise and (**b**) multiplicative noise.

Following the standard digital-image degradation models described in Ref. [42], let E(u,v) denote the real-valued encrypted distribution obtained from Equation (5) on the numerical scale used in the simulations. No additional normalization is applied to E(u,v) before introducing the noise perturbation. The encrypted distributions affected by additive and multiplicative noise are respectively defined as $E_{A}\left(u,v\right)=E\left(u,v\right)+\eta_{A}\left(u,v\right)$ and $E_{M}\left(u,v\right)=E\left(u,v\right)\left[1+\eta_{M}\left(u,v\right)\right]$, where $\eta_{A}\left(u,v\right)$ and $\eta_{M}\left(u,v\right)$ are zero-mean Gaussian white-noise distributions with variances $\sigma_{A}^{2}$ and $\sigma_{M}^{2}$, respectively. The additive model introduces a signal-independent perturbation, whereas in the multiplicative model the noise contribution is scaled by the local value of the encrypted distribution. Figure 4 shows the decrypted images obtained when the encrypted image of Figure 2f is degraded by noise. The applied perturbation is zero-mean Gaussian white noise with variance $\sigma^{2}=0.2$. Figure 4a presents the decrypted image corresponding to additive noise, whereas Figure 4b corresponds to multiplicative noise. In both cases, decryption is performed with the correct values of the seven security keys. The NRMSE values between the primary image of Figure 2a and the decrypted images of Figure 4a and Figure 4b are 0.2442 and 0.2393, respectively. For this noise test, the decrypted images obtained exhibit very similar visual quality.

The variance $\sigma^{2}$ of the zero-mean Gaussian white noise was also progressively increased to determine the authentication-failure thresholds for additive and multiplicative noise. These thresholds, defined as the lowest tested variances at which no text could be extracted from the recovered QR code using a smartphone camera, were found at 0.73 and 0.77, respectively. The corresponding NRMSE values between the original primary image f(x,y) and the corresponding decrypted images $\hat{f}\left(x,y\right)$ were 0.5873 and 0.5694. At each identified variance threshold, the recovered primary image did not preserve sufficient QR-code information for text extraction; consequently, the simultaneous authentication of the three images failed.

Despite the quality loss of Figure 3c,d and Figure 4a,b, the primary image of Figure 2a remains recognizable in all cases. When these images are read as QR codes, the retrieved message is, in every case, *The authentication process has been successful*. Therefore, simultaneous authentication of the three images, $p_{1}\left(x,y\right)$, $p_{2}\left(x,y\right)$, and $p_{3}\left(x,y\right)$, is achieved even when the encrypted image is degraded by noise or partial occlusion. These results show the robustness of the proposed encryption–decryption system and authentication process under the evaluated degradation levels affecting the encrypted image.

## 4. Conclusions

A nonlinear JTC-based encryption system in the FD for the simultaneous authentication of three images has been presented. The proposed security system combines one real-valued encrypted image, four RPMs, and three images to authenticate into a seven-key security architecture, thereby extending previous nonlinear authentication approaches toward a more demanding multi-image validation framework. The main contribution of this work lies in the two new nonlinear modifications introduced into the JPS, based on the subtraction of two specific intensity distributions and on a three-component normalization term, which strengthen ciphertext protection, require the simultaneous consistency of all seven security keys for correct primary-image recovery, and reinforce resistance against the established direct-inversion and plaintext-oriented attack procedures previously analyzed for nonlinear JTC encryption systems. Unlike the previous two-image simultaneous authentication system, the proposed architecture cannot be obtained by merely incorporating a third authentication distribution, because such a direct extension generates additional mutual JPS terms that preclude the correct recovery of the primary image during decryption, even when all security keys match. To overcome this limitation, the encryption and decryption algorithms were reformulated through two new nonlinear modifications of the JPS. These modifications ensure the exact cancellation of the complete intensity factor associated with the three authentication distributions during correct-key decryption, thereby enabling the correct recovery of the primary image and the simultaneous authentication of the three images. Therefore, the proposed system constitutes a necessary algorithmic and mathematical reformulation of the previous two-image approach rather than a direct numerical extension from two to three authentication images. The numerical results confirm that, when the seven correct security keys are used, the primary image is recovered with high fidelity and the embedded QR code provides successful simultaneous authentication of the three images due to the two new nonlinear modifications introduced into the JPS, whereas an incorrect key produces severe degradation in the decrypted image and prevents valid authentication. In addition, the extremely large random cryptographic key space associated with the four statistically independent RPMs provides strong resistance against exhaustive brute-force attacks under the adopted sampling and quantization model. From a systems perspective, the main advantage is the fusion of cryptographic validation and authentication into a single decryption workflow. This coupled behavior establishes a deterministic joint-validation condition, because successful reconstruction requires all seven security keys to match their encryption-stage counterparts simultaneously. Furthermore, the information encoded in the recovered QR image provides a semantic validation criterion, because successful authentication is determined by the recovery of the expected QR message rather than by exact pixel-by-pixel equality between the primary and decrypted images. The robustness analysis performed under partial occlusion and under additive and multiplicative zero-mean Gaussian white noise affecting the encrypted distribution shows that the proposed decryption–authentication system preserves recognizable primary-image recovery and successful QR-based validation under moderate degradation conditions. The extended tests further identify the lowest evaluated degradation levels at which no text can be extracted from the recovered QR code: an occluded area of 37%, an additive-noise variance of 0.73, and a multiplicative-noise variance of 0.77. Overall, the reported numerical results support the numerical feasibility of the proposed nonlinear JTC-based optical encryption architecture for the simultaneous authentication of three images under the assumptions and operating conditions considered in this work. Experimental optical validation, evaluation under practical image-acquisition variability, and comprehensive architecture-specific cryptanalysis remain subjects for future work.

## Data Availability

The retinal and fingerprint images used in this work were acquired directly by the authors from a single member of the author group and are not part of a public or third-party dataset. The processed images used in the numerical simulations are presented in Figure 2. The original biometric images are not publicly available to protect the privacy of the author who provided them.

## Abbreviations

The following abbreviations are used in this manuscript:

DRPE	Double Random Phase Encoding
FD	Fourier Domain
FT	Fourier Transform
JPS	Joint Power Spectrum
JTC	Joint Transform Correlator
NRMSE	Normalized Root Mean Square Error
QR	Quick Response
RPMs	Random Phase Masks
